# Supplementary figures and images for: Quantifying murine placental extracellular vesicles across gestation and in preterm birth data with tidyNano: A computational framework for analyzing and visualizing nanoparticle data in R
Source: PLoS One. 2019 Jun 18;14(6):e0218270. doi: 10.1371/journal.pone.0218270 (PMC6581270; doi:10.1371/journal.pone.0218270)

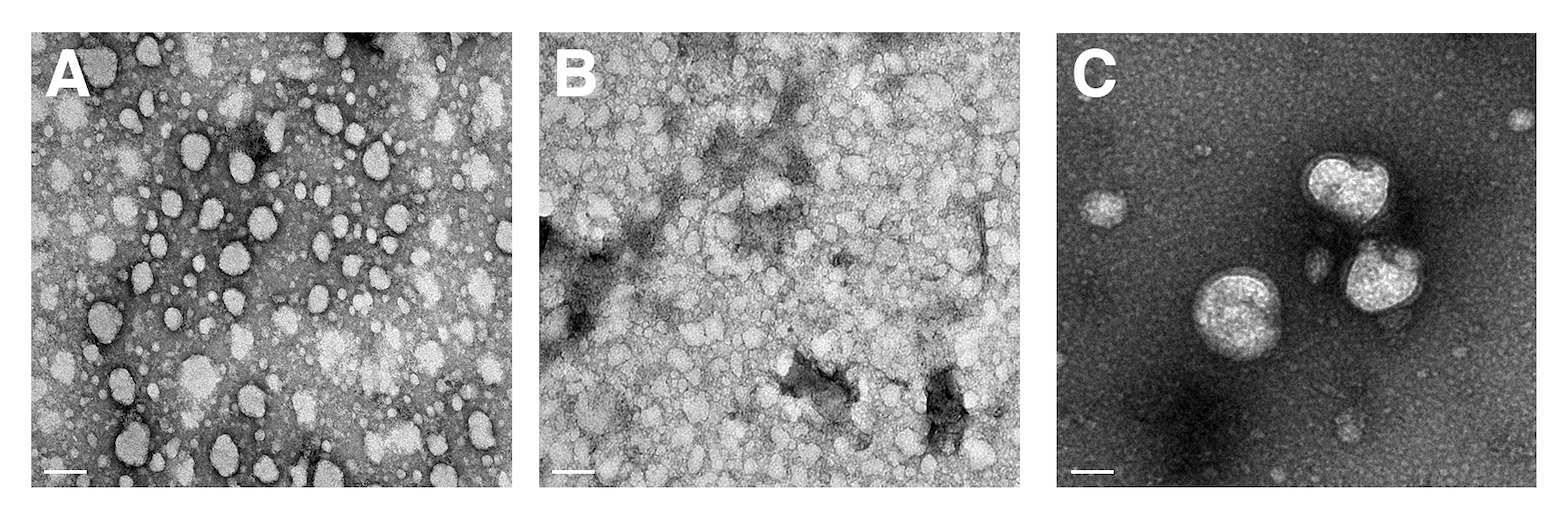

Supplement: S1 Fig — Representative electron micrographs of plasma exosomes isolated by Total Exosome Isolation reagent of (A) non-pregnant and (B, C) GD14.5 exosomes. Scale bar represents 100nm. (TIF) [file pone.0218270.s001.tif]

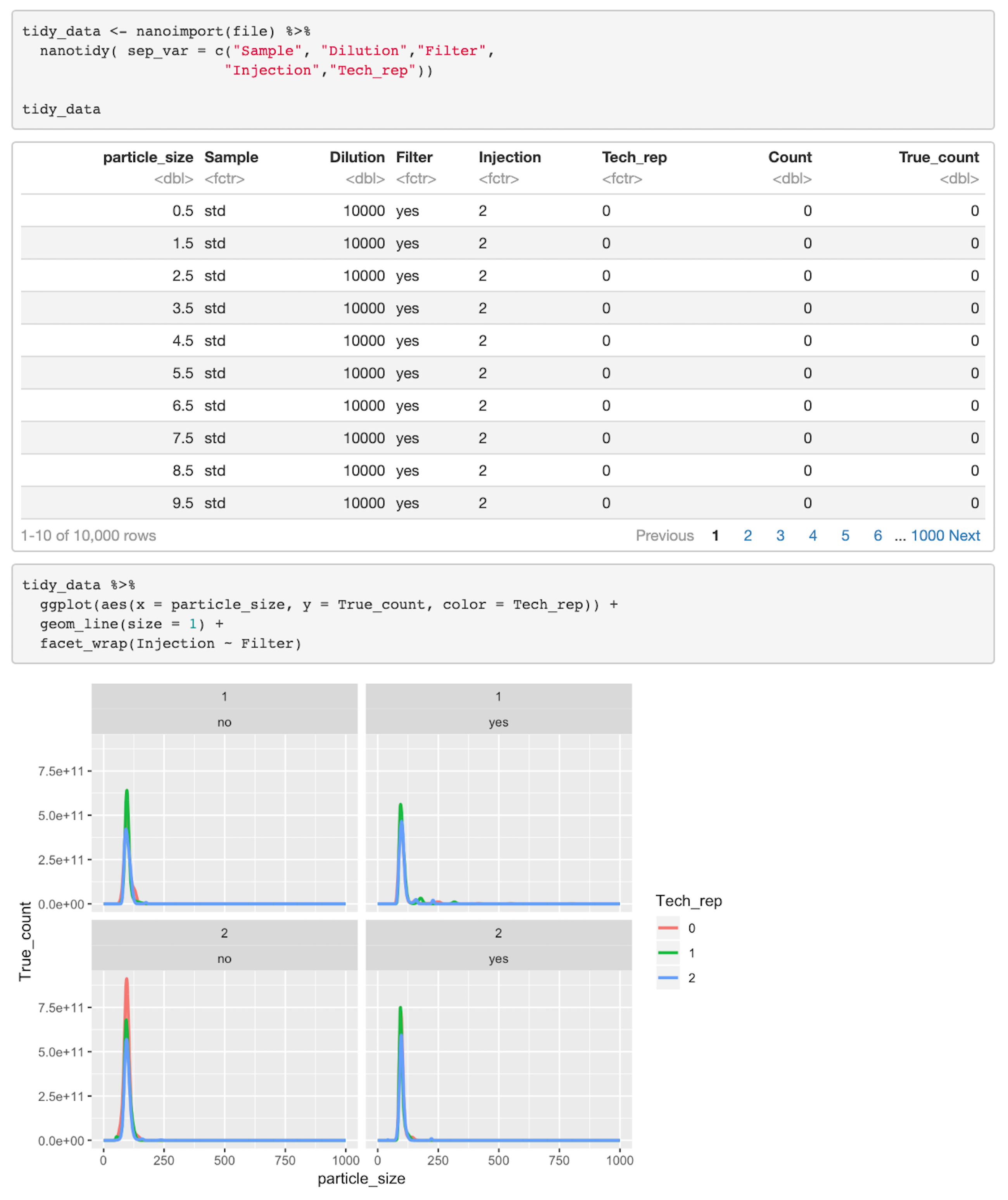

Supplement: S2 Fig — Sample workflow of importing data into R using tidyNano functions. (TIF) [file pone.0218270.s002.tif]

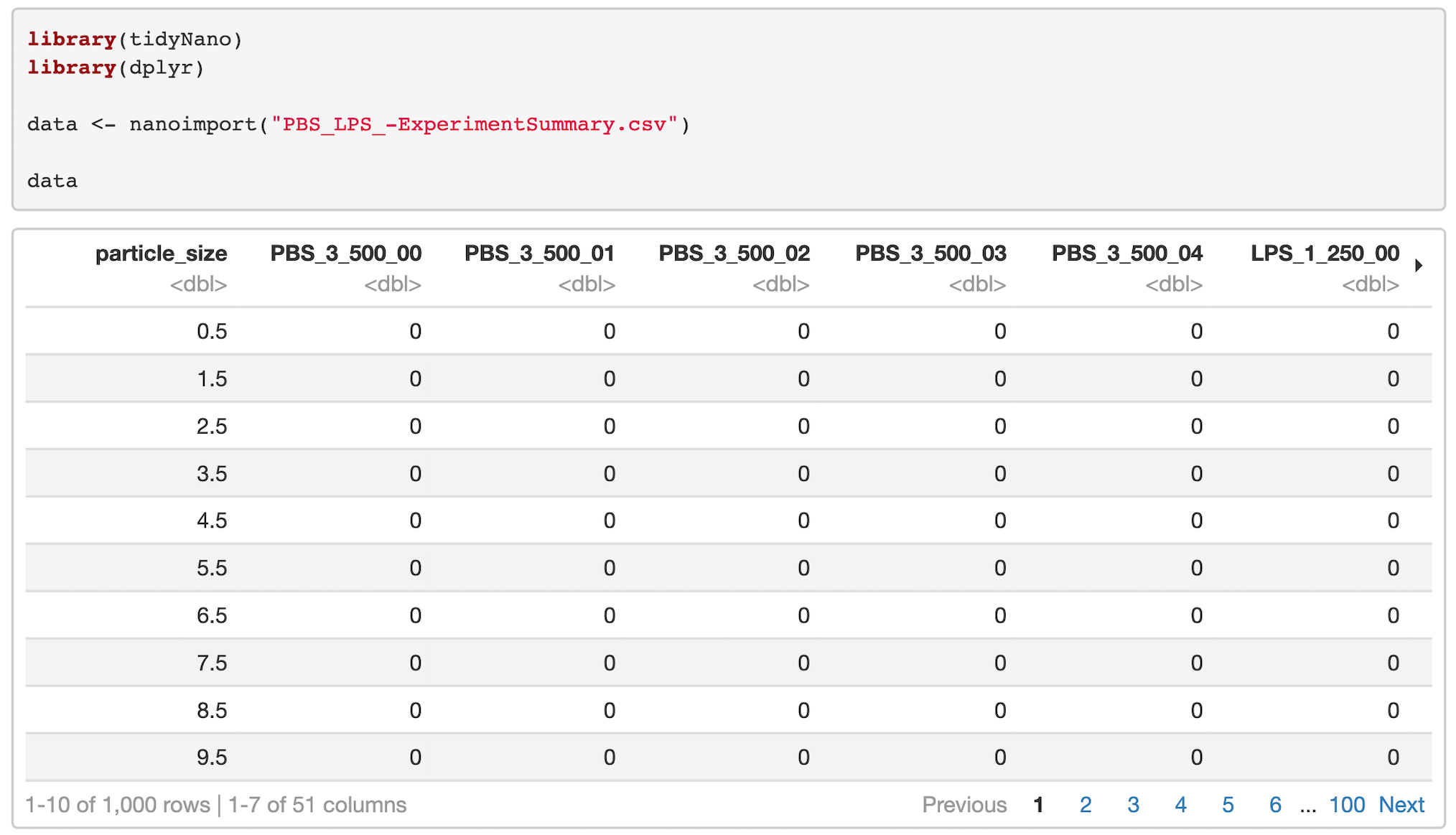

Supplement: S3 Fig — Raw count data from NTA .csv files can be extracted and imported into the R environment with the nanotidy() function. (TIF) [file pone.0218270.s003.tif]

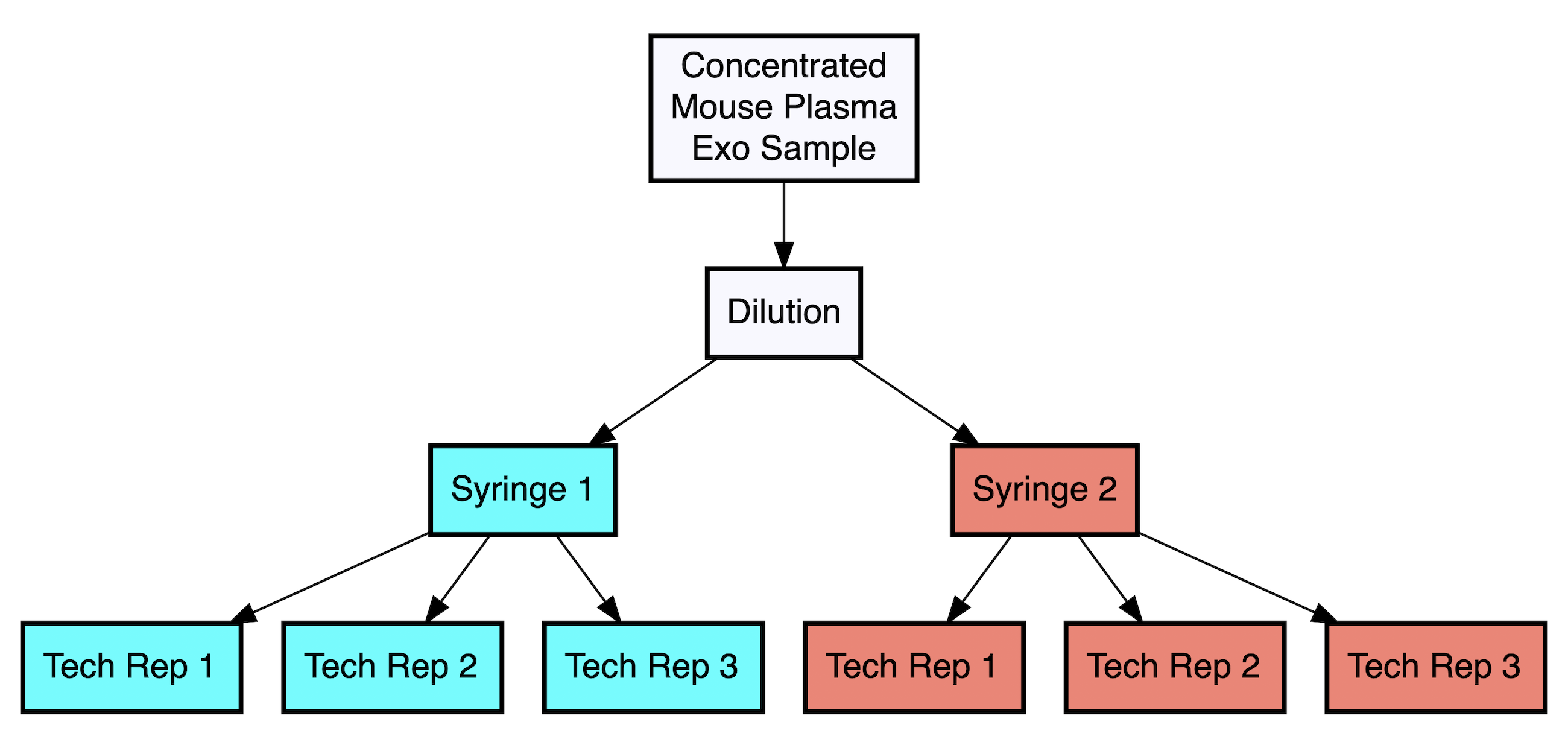

Supplement: S4 Fig — Each plasma exosome sample was diluted, separated into two separate syringes, and was measured by NanoSight through recording of three 30-second videos. (TIF) [file pone.0218270.s004.tif]

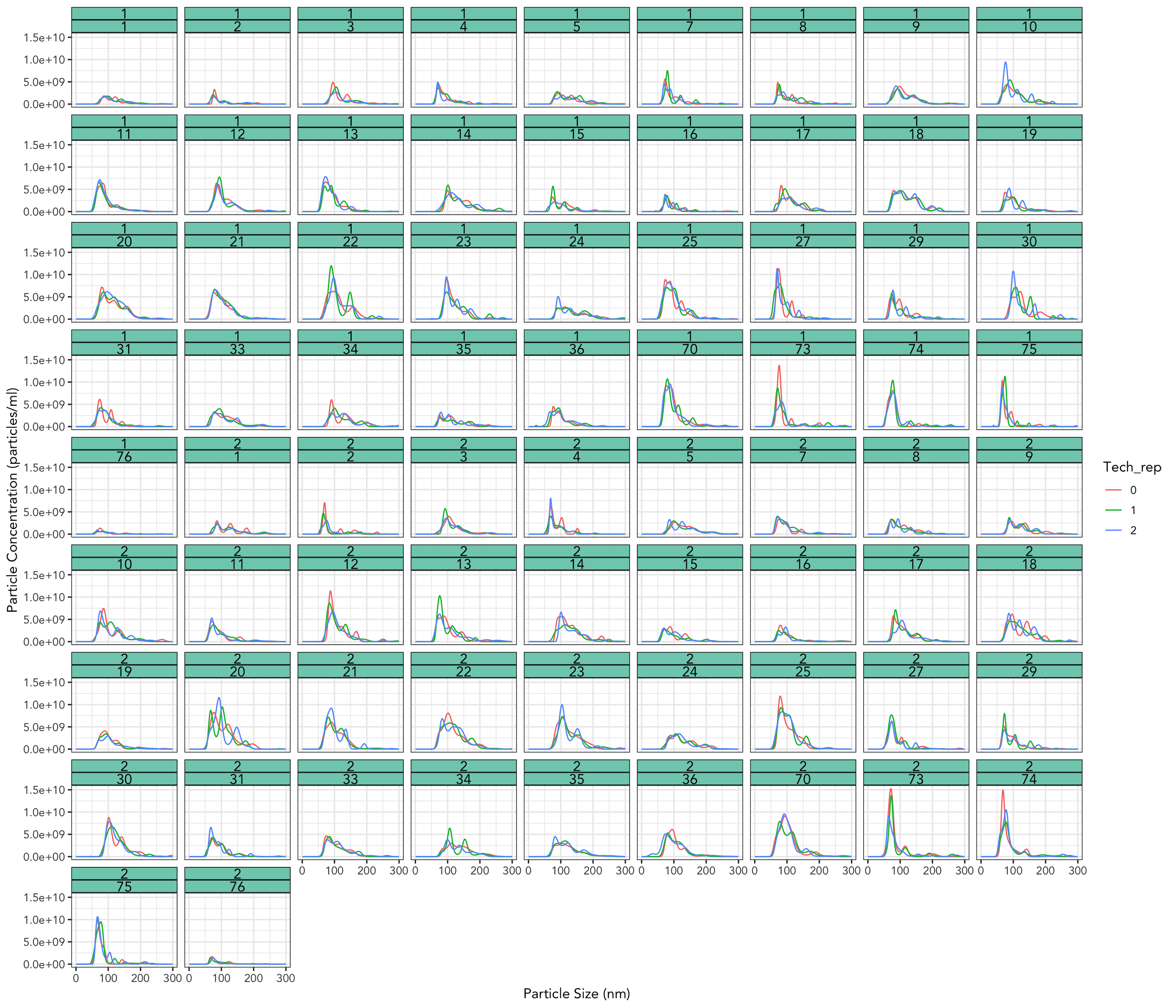

Supplement: S5 Fig — Faceted plot of plasma exosome size and particle concentration of all samples (n = 76) in experimental study where each sample was tested twice with three technical replicates. (TIF) [file pone.0218270.s005.tif]

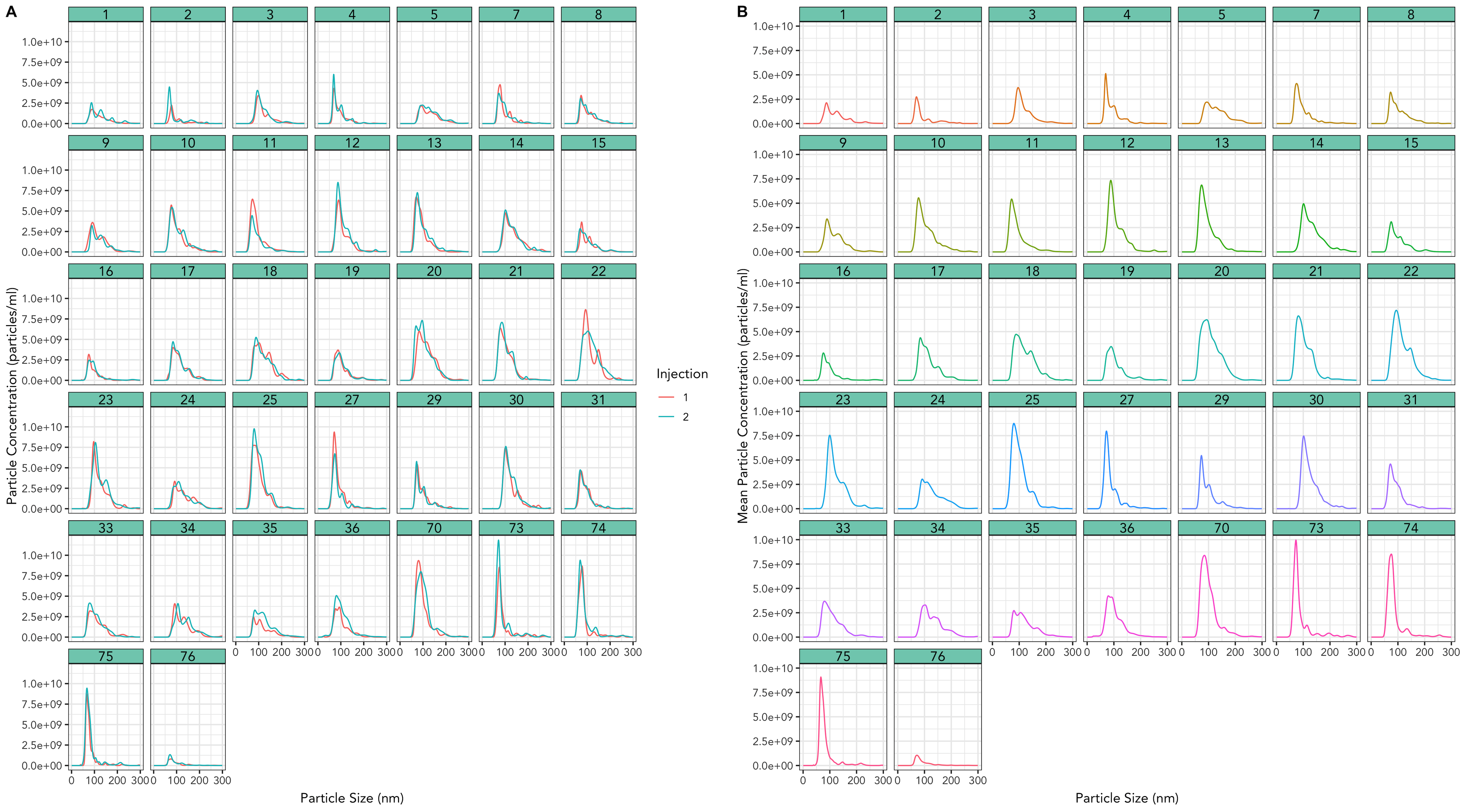

Supplement: S6 Fig — Each summary output of the nanolyze() aggregation function can be visualized. (A) Plot of sample mean particle concentration from three technical replicate measurements (n = 76). (B) Plot of mean injection data from two syringe injection measurements (n = 76). (TIF) [file pone.0218270.s006.tif]

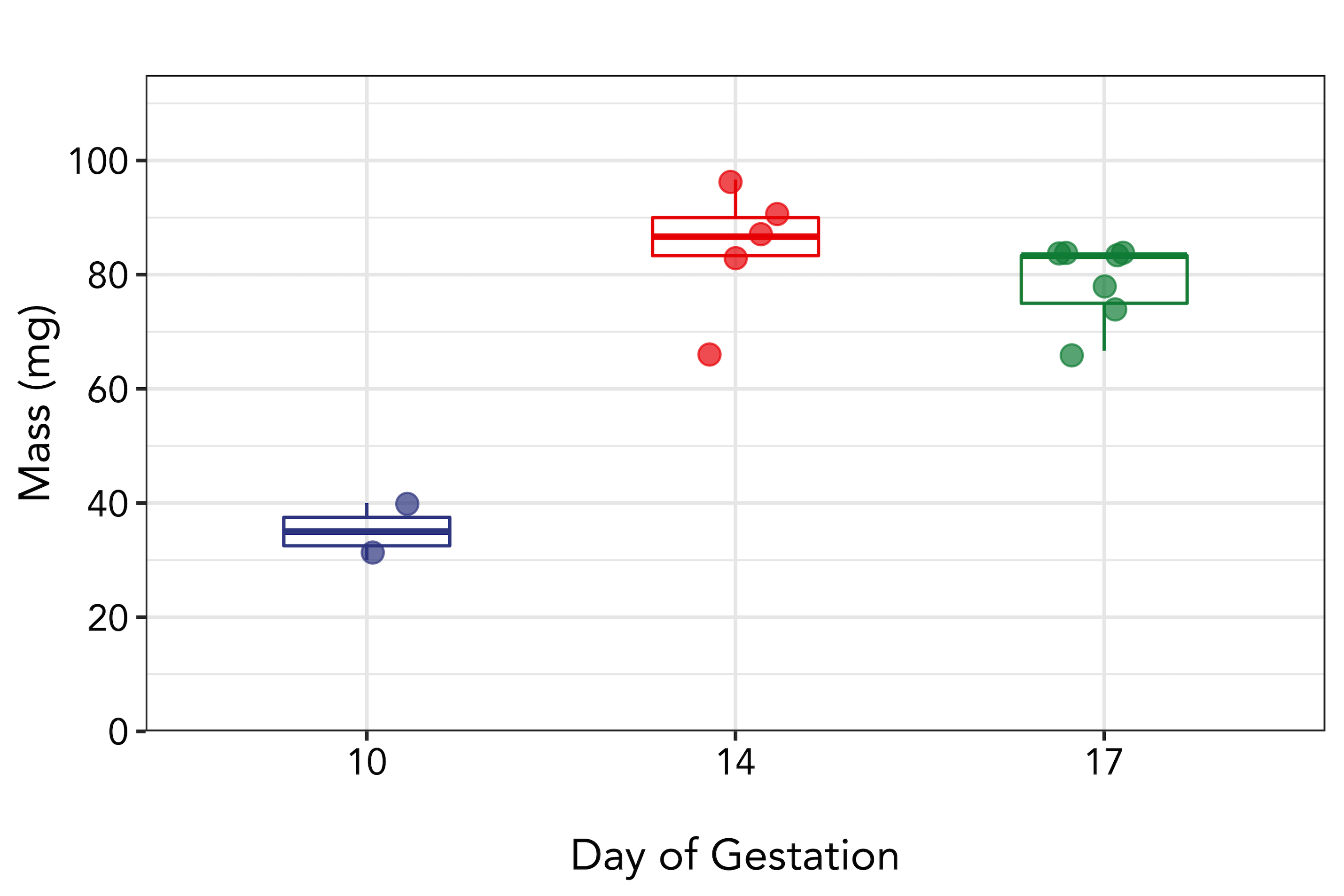

Supplement: S7 Fig — Placental mass across gestation in WT mated C57B/6 mice. Points represent individual placentas. (TIF) [file pone.0218270.s007.tif]
