# Supplementary material for: Quantifying murine placental extracellular vesicles across gestation and in preterm birth data with tidyNano: A computational framework for analyzing and visualizing nanoparticle data in R
Source: PLoS One. 2019 Jun 18;14(6):e0218270. doi: 10.1371/journal.pone.0218270 (PMC6581270; doi:10.1371/journal.pone.0218270)
Supplement: S8 Fig — (PDF) [file pone.0218270.s008.pdf]

NANOSIGHT

74_500_1_

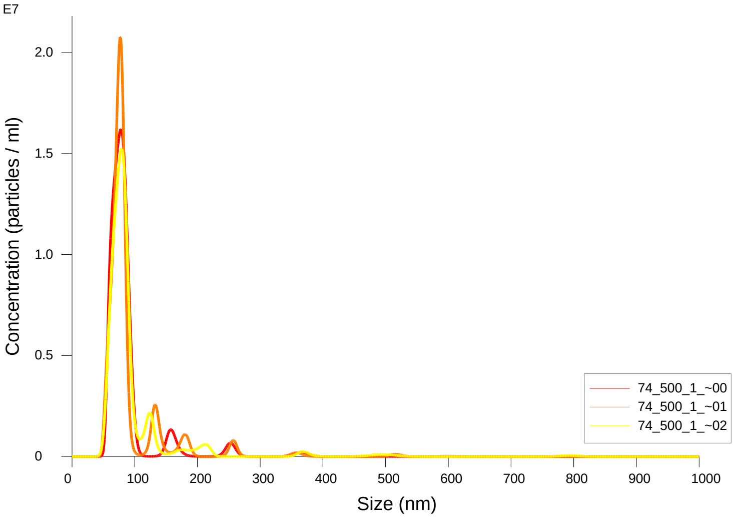

FTLA Concentration / Size graph for Experiment:
74_500_1_

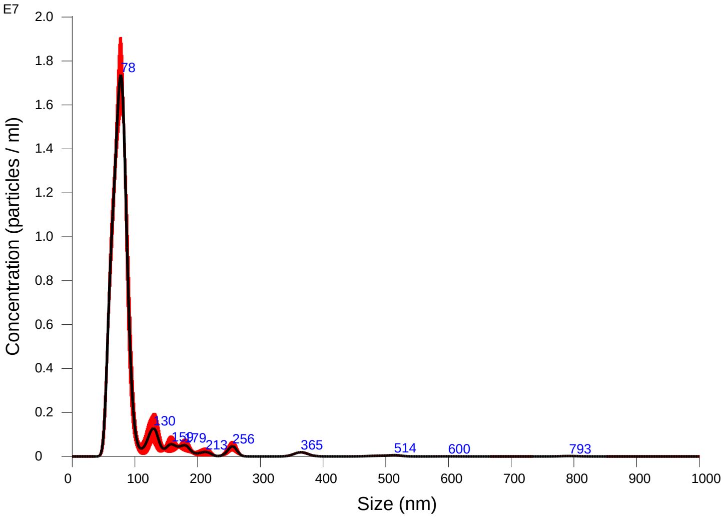

Averaged FTLA Concentration / Size for Experiment:
74_500_1_
Error bars indicate + / -1 standard error of the mean

Included Files

74_500_1_00
74_500_1_01
74_500_1_02

Details

NTA Version:	NTA 3.2 Dev Build 3.2.16
Script Used:	SOP Standard Measurement 11-35-16AM 21Mar2017.txt
Time Captured:	13:11:36 21/03/2017
Operator:	SN
Pre-treatment:	
Sample Name:	SJ_74 1:500 in PBS no filter
Diluent:	PBS
Remarks:	installation with 488 laser SJ_74 1:500 in PBS no filter

Capture Settings

Camera Type:	sCMOS
Laser Type:	Blue488
Camera Level:	12
Slider Shutter:	1200
Slider Gain:	146
FPS	25.0
Number of Frames:	749
Temperature:	22.7 ºC
Viscosity:	(Water) 0.938 - 0.938 cP
Dilution factor:	Dilution not recorded
Syringe Pump Speed:	50

Analysis Settings

Detect Threshold:	4
Blur Size:	Auto
Max Jump Distance:	Auto: 15.3 - 16.1 pix

Results

Stats: Merged Data

Mean:	91.1 nm
Mode:	77.4 nm
SD:	57.0 nm
D10:	60.2 nm
D50:	76.5 nm
D90:	129.3 nm

Stats: Mean +/- Standard Error

Mean:	91.1 +/- 2.0 nm
Mode:	77.9 +/- 0.6 nm
SD:	56.3 +/- 6.7 nm
D10:	60.2 +/- 0.2 nm
D50:	76.6 +/- 0.7 nm
D90:	119.9 +/- 11.2 nm
Concentration (Upgrade):	5.56e+008 +/- 3.87e+006 particles/ml
	68.7 +/- 0.6 particles/frame
	68.7 +/- 0.4 centres/frame

NANOSIGHT

74_500_1_

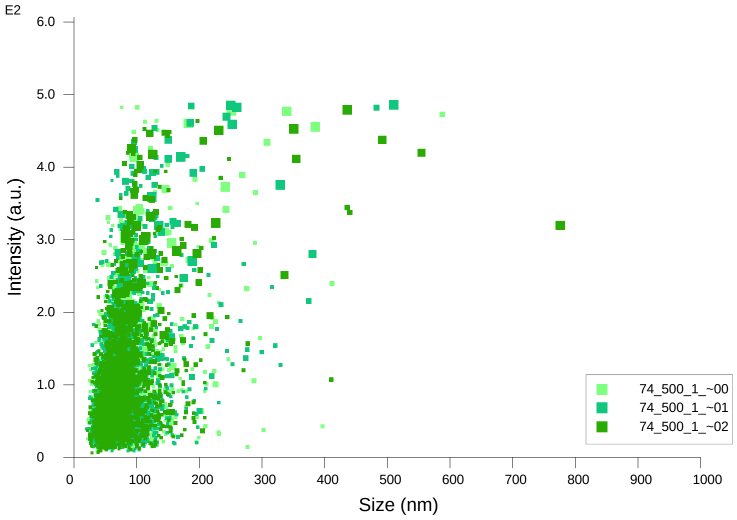

Intensity / Size graph for Experiment:

74_500_1_
